# Is it worth it?: The experiences of persons with multiple sclerosis as they access health care to manage their condition

**DOI:** 10.1111/hex.13109

**Published:** 2020-07-22

**Authors:** Julie Pétrin, Catherine Donnelly, Mary‐Ann McColl, Marcia Finlayson

**Affiliations:** ^1^ School of Rehabilitation Therapy Queen's University Kingston ON Canada

**Keywords:** health‐care access, health‐care seeking, multiple sclerosis, patient‐centred care, qualitative

## Abstract

**Background:**

People with multiple sclerosis (MS) require complex care throughout life. Canadians with MS are high users of health‐care services, yet still report unmet health‐care needs and low satisfaction with services received.

**Objective:**

This study aimed to investigate the health‐care access experiences of Ontarians with MS as they manage their condition.

**Design and Participants:**

Interpretive description guided data collection and analysis. Forty‐eight people living across seven communities participated. Thirty‐eight participated in one of five focus groups; the remaining 10 participated in an individual semi‐structured interview.

**Results:**

Participants described the experience of accessing care as a decisional process, guided by a form of cost‐benefit analysis. The process determined whether seeking conventional health‐care services ‘is worth it’. Most participants felt that the energy and resources required to access the health‐care system outweighed their expected outcomes, based on past experiences. Participants who did not see the benefit of care seeking turned to self‐treatment, use of complementary and alternative services, and engaged in patterns of health‐care avoidance until a crisis arose.

**Discussion and Conclusion:**

Findings suggest that a renewed effort to promote patient‐centred care and a biopsychosocial approach may improve the health‐care access experiences of persons with MS and reduce service avoidance.

## 1 INTRODUCTION

Multiple sclerosis (MS) is a chronic neurodegenerative condition, affecting the central nervous system. Common symptoms are fatigue, pain, spasticity, mobility and balance impairments, incontinence and sexual dysfunction^1^. MS is one of the leading causes of non‐traumatic neurological disability in young adults in North America^2^. The prevalence rate in Canada is 290/100 000, one of the highest in the world^3^. MS has an unknown aetiology, an unpredictable course and symptomology, and no available cure. Together, these factors make MS difficult to manage, contributing to the need for complex care throughout life^4^, ^5^.

Researchers report high use of health‐care services among individuals with chronic illnesses^6^, ^7^, ^8^, and Canadians with MS rank among the highest^9^, ^10^. Annually, Canadians with MS visit their family physician nearly twice as often^10^, are admitted to hospital 2.5 times more and consult mental health professionals 2.6 times more than age‐ and sex‐matched peers^9^. Nevertheless, they report unmet health needs, poor health ratings and low satisfaction with health‐care services^11^, ^12^, ^13^. International research has uncovered a link between these outcomes and poor access to healthcare within the MS population^14^, ^15^, raising questions about the health‐care access experiences of Canadians with MS. This international research used a biopsychosocial approach, which recognizes the combined impact of biological, psychological and social factors on an individual's health and wellbeing^16^. Therefore, using this approach may offer insights into the experiences of access to health care among persons with MS in Canada.

‘Access to health care’ is a complex multidimensional process that includes an individual's path to care seeking, their point of entry into the health‐care system, and their use of services within that system^17^. In health service research, investigation of access tends to focus on the point of entry and use of services, considering issues of utilization, supply and demand, availability, accessibility, affordability and acceptability^18^, ^19^, ^20^. Within the social sciences, investigations of access tend to focus on the social, cultural, psychological and behavioural factors that influence an individual's path to care seeking and point of entry^21^, ^22^, ^23^, ^24^. We choose to use a combined perspective, considering access to healthcare as spanning from the path to care seeking through to actual use of services^25^, ^26^. Using this perspective, and a biopsychosocial approach, we investigated the health‐care access experiences of Canadians with MS as they manage their condition.

## 2 METHODS

We used interpretive description to inform all aspects of this study's design. This applied qualitative methodology aligns with a constructivist approach and focuses on knowledge generation to inform clinical practice^27^. Initial data collection involved five focus groups (FG). To explore emerging concepts, we added 10 semi‐structured telephone interviews (TI) with participants from communities in northern Ontario. These interviews allowed in‐depth exploration of access issues related to rural geography and self‐management. Recruitment, data collection and analysis occurred between November 2017 and April 2018.

### 2.1 Participants and recruitment

The Health Science Research Ethics Board of the researchers' university granted ethical approval for the study. We purposefully recruited focus group participants from five communities of different sizes. We called 21 persons with MS who had previously consented to be re‐contacted by our research team. We also distributed study information through the MS Society's research portal, social media platforms, local chapters' email lists, support groups and educational events, and by distributing flyers in local communities.

Focus group participants were screened to ensure that they met the following criteria: (a) self‐reported having a definite diagnosis of MS from a neurologist; (b) at least 18 years of age; (c) able to tolerate a 90‐minute discussion; (d) able to communicate in English; (e) and able and willing to attend one focus group.

For the telephone interviews, we recruited persons with MS living in rural communities in Northern Ontario (≥3 hours from tertiary care) through the Ontario Division of the MS Society of Canada. Potential participants were screened to the criteria above, with the following revisions: (3) able to tolerate a 60‐minute discussion (5) by telephone.

### 2.2 Data collection

Participants completed a self‐reported questionnaire to capture demographic, clinical and information about health service use before the session. The first author facilitated all focus groups, after receiving training.

All focus groups had a co‐facilitator who provided logistical support. The focus groups ranged from 5‐12 participants and lasted between 90‐110 minutes. The sessions were facilitated using a semi‐structured interview guide. To ensure that the interview guide would elicit a full range of experiences about accessing health‐care services, it was informed by previous MS access research^5^, ^28^ and health‐care access theory grounded in a biopsychosocial approach^25^, ^26^. The guide was piloted with five individuals with MS to ensure clarity and relevance prior to use and was adjusted iteratively to reflect emerging concepts and themes (eg health‐care provider communication; preconceptions of outcomes). For exemplar questions from the guide, see Table 1.

**TABLE 1:** Exemplar questions from interview topic guide

How do you know when you need to seek care for your MS management issue?
Once you reach a tipping point, how do you decide where to get help and from whom?
Describe your experience of interacting with healthcare providers regarding an MS management issue?
When you are with your healthcare provider and a management issue is raised, describe how a decision is made to address them. How do you feel about how these decisions are made?
How do you feel about the services and healthcare providers available to address your MS management issues?
What do you think are the strengths and weaknesses of your current healthcare in managing your MS?
Please describe what perfect access to healthcare means to you.

The first author conducted individual interviews by telephone, which lasted 51‐116 minutes. The same semi‐structured guide was used; however, additional follow‐up questions were added to gain more in‐depth information about certain concepts (eg rural living, self‐management, delayed care seeking). Focus groups and telephone interviews were recorded and professionally transcribed verbatim. We verified the transcripts against the audio recordings to ensure accuracy and de‐identified them using pseudonyms.

### 2.3 Data analysis

Analysis began immediately after the first focus group. Concurrent collection and analysis allowed for comparisons of concepts and ideas between focus groups and interviews, using constant comparison analysis^29^, consistent with interpretive description methodology^27^.

The analysis process involved the first author reading and re‐reading the transcripts to become familiar with the data. During this process, we continually questioned the data, searching for similarities and differences across ideas, perceptions, attitudes and experiences. Open coding was initiated with the assistance of the focus group co‐facilitators and an additional arms‐length individual. All individuals coded independently and then met to discuss and explore interpretations. The first and senior author met on a weekly basis to discuss the ongoing coding process.

The initial open codes were examined for patterns and relationships across incidents and sessions, allowing coding to become more specified. These narrower codes were examined for similarities and differences, which allowed for clearer understanding of the categories and themes. However, consistent with interpretive description no theoretical framework was ascribed to the data^27^, rather the data were interpreted through a biopsychosocial lens. We re‐contextualized and organized the findings to illuminate the essence of the health‐care access experiences of participants. We deepened the overall analytic process by using the sorting and query functions of the data management programme, ATLAS.ti^30^.

### 2.4 Establishing rigour

We invited all focus group participants to be a part of the member‐check process. Thirteen accepted the invitation and received a summary of the group discussion for input. They did not suggest any major changes. The first author also kept an analytic and reflexive journal throughout the study. All meetings, notes and coding steps were kept, maintaining a robust audit trail. To ensure the credibility of the findings, triangulation of data sources (interviews and focus groups) and researchers (multiple individuals on analysis team) was employed. The findings are presented with contextual information to inform the readership of the analytic logic.

### 2.5 Findings

#### 2.5.1 Participant demographics

Participants were recruited primarily through local MS chapter personnel (n = 23) and word of mouth (n = 11), followed by local MS events (n = 4), previous participant list (n = 6), social media (n = 4) and flyers (n = 2). Everyone who was interested met the eligibility criteria, although two could not attend the focus group. A final forty‐eight participants shared their experiences on accessing health‐care services in managing their MS. Participants were 49.6 years of age (SD: 12.3; range: 27‐71) on average and had lived with MS for an average of 15.0 years (SD: 10.5; range: 3‐42). Most were female (n = 32, 67%), almost all identified as Caucasian (n = 45, 94%), and just under half reported having relapsing‐remitting MS (n = 22, 46%). All but one participant had a regular family physician and all participants had a neurologist. Most of the participants (n = 38, 79%) saw their neurologist at an MS specialty clinic. Just under two‐thirds of participants (n = 29, 60%) considered their main source of care to be their neurologist, while 31% (n = 15) selected their family physician. Persons with MS reported visiting their neurologist an average of 1.5 times a year (SD: 1.1) and their family physicians 3.8 times a year (SD: 3.9). For further socio‐demographic characteristics, refer to Table 2. For further MS‐related characteristics and health service use, refer to Table 3.

**TABLE 2:** Socio‐demographic characteristics (N = 48)

Variables	n (%)
Marital Status	
Married/Partnership	33 (68.8)
Separated/Divorced	8 (16.6)
Single	7 (14.6)
Living Arrangements	
Alone	11 (22.9)
Spouse/Partner	22 (45.8)
Spouse/Partner and Child(ren)	10 (20.8)
Parents or Siblings or Child(ren)	5 (10.4)
Long‐term care home	1 (2.1)
Employment Status	
Unable to work/Disability	24 (50.0)
Employed full‐time	11 (22.9)
Employed part‐time	4 (8.3)
Retired	9 (18.8)
Household Income (CAD)	
Less than 30 000	10 (21.3)
30 000‐59 999	12 (25.5)
60 000‐89 000	12 (25.5)
90 000 or above	10 (20.8)
I'd rather not say	3 (6.4)
Education	
High School	2 (4.3)
College/Vocational	22 (45.8)
Bachelors and Masters	23 (48.9)
Residential	
Large City (>100 000)	29 (61.7)
Medium city (50 000‐100 000)	4 (8.5)
Smaller city (10 000‐49 999)	7 (14.9)
Out in the country (<10 000)	7 (14.9)

**TABLE 3:** MS‐Related Characteristics and Health Service Use Information (N = 48)

Variables	n (%)
Type of MS	
Relapsing‐Remitting MS	22 (45.8)
Secondary Progressive MS	10 (20.8)
Primary Progressive MS	12 (25.0)
Primary Relapsing MS	1 (2.1)
Unknown	3 (6.3)
Taking DMT^a^	
Yes	17 (35.4)
No	29 (60.4)
Not sure	2 (4.2)
PDSS Score^b^	
0‐2 (Mild disability)	13 (27.1)
3‐5 (Moderate disability)	20 (41.7)
6‐8 (Severe disability)	15 (31.3)
Main source of MS Management	
General Practitioner (GP)	15 (31.3)
Neurologist	29 (60.4)
Physiotherapist	2 (4.2)
Nurse	2 (4.2)
Where do you receive most MS Care	
MS Clinic	22 (45.8)
Neurologist in Hospital	5 (10.4)
GP/Private Clinic	5 (10.4)
Interprofessional Team Clinic	3 (6.3)
Walk In/Afterhours	3 (6.3)
Emergency	2 (4.2)
Rehabilitation Centre	1 (2.1)
Other	7 (14.6)

^a^ DMT: Disease‐modifying therapy.

^b^ PDSS: Patient Determined Disease Steps, which is a self‐reported measure of disability in persons with MS.

#### 2.5.2 Overview of findings

**FIGURE 1:**
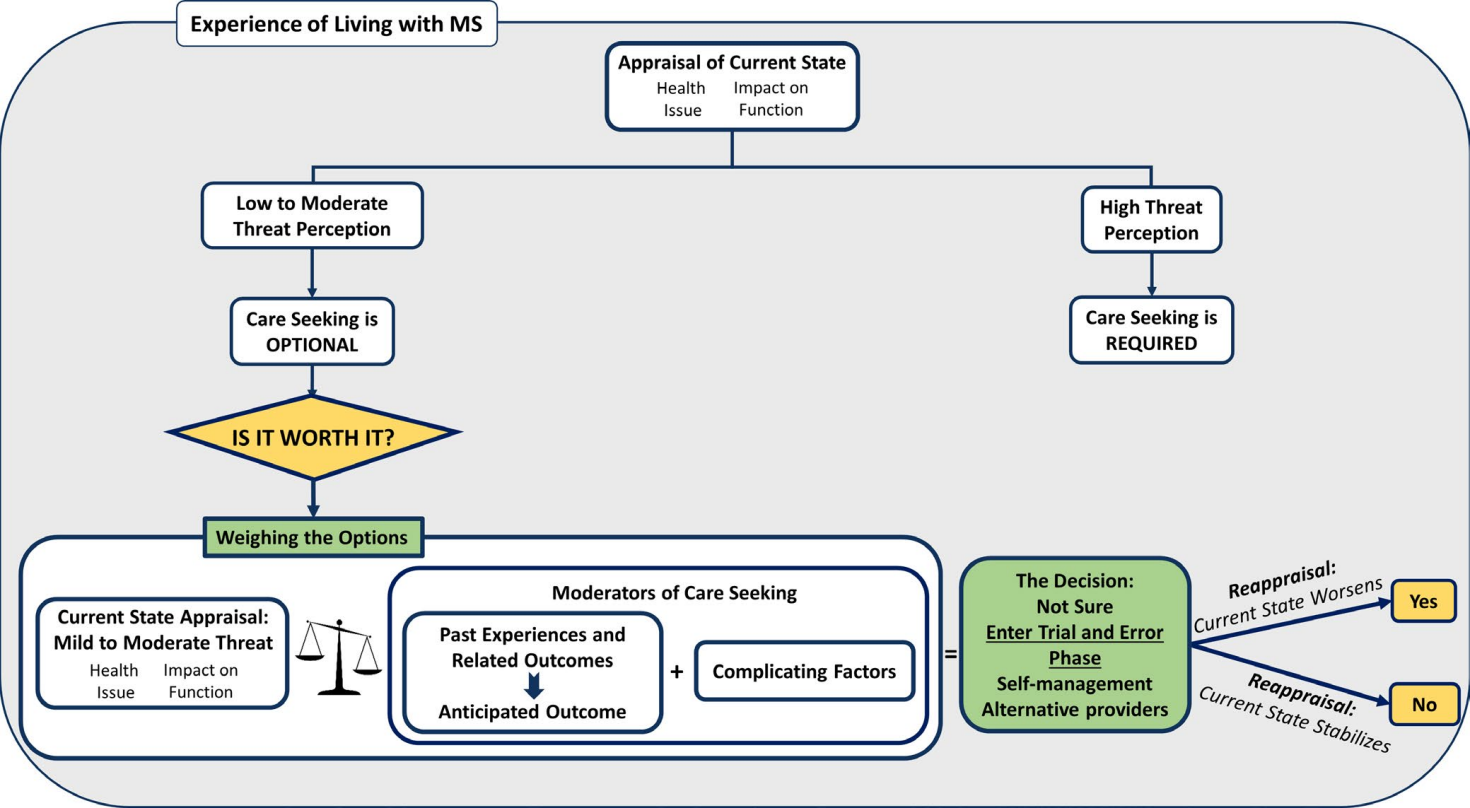
Conceptual model depicting persons with MS' experience of accessing health‐care services to manage their condition

Participants' experiences of accessing care to manage their condition revolved around the process of seeking care and the factors that impacted this process. Past experiences of engaging with the conventional health‐care system, defined by participants as including hospitals, clinics and emergency departments employing doctors, nurses and/or specialists, were the most important factor. Participants were using a form of cost‐benefit analysis to decide whether seeking conventional health‐care services was worth it. The question ***‘Is it worth it?’*** became the overarching analytic theme. Other themes reflected the process participants engaged in to answer this question. This section provides an overview of the themes and their relationships (see Figure 1 for the conceptual model of the themes).

Participants began the process of accessing health care by engaging in an *appraisal of their current state,* prompted by a potential MS‐related health issue. This appraisal was then weighed against reflections of their *past experiences* and *related outcomes,* as well as multiple *complicating factors,* which were taken into account to come to *a final decision* about whether accessing health care was worth it.

This decisional process was impacted by the person's experience of living with MS over time. Participants who had lived with MS for many years discussed the concepts of symptom normalization, as well as a growing repertoire of past experiences with self‐management and health‐care services to aid their decision making. The steps in the decisional process (themes and subthemes) are elaborated below.

##### Appraisal of current state

When a new potentially MS‐related health issue arose, participants appraised their current state based on the severity, duration, progression and nature of the health issue. Across all responses, the leading causes of care seeking were pain, falls, fatigue and uncertainty about whether the health concern was MS‐related. A key component of appraisal was how much the health issue impacted their ability to fully participate in daily life:

[You seek care] when it's affecting your personal life and what you used to do, you're not able to do, anymore. (Salinda, FG2)

Appraisal of the health issue also involved an evaluation of its potential threat. Appraisals led to one of two main outcomes: (a) health‐care seeking as a requirement or (b) health‐care seeking as optional.

In cases where individuals felt they were in a state of ‘crisis,’ their decisional process ended, as health‐care seeking became a requirement. However, individuals who did not feel threatened or in a state of crisis (low to moderate threat) continued their decisional process:

I usually don't seek out a lot of intervention, unless I feel really threatened in my situation. (Terry, FG3)

One participant described normalizing a health issue and waiting until it has progressed significantly before feeling the need to attempt accessing care:

[…] It's very easy to minimize them [MS issues]. […] I need to be pretty convinced that, um, it's a relapse or, um, it's a significant symptom, to actually seek out care. (Alexis, TI)

The answer to the question ‘Is it worth it?’ was also described as ongoing, as participants engaged in reappraisal of their current state on a continual basis. If at any time a reappraisal leads to a high threat perception, individuals described care seeking as a requirement.

#### 2.5.3 Factors moderating care seeking

When health‐care seeking was deemed optional, they continued the decisional process to determine if accessing care was worth it, by taking into account past experiences, outcomes of care seeking and other complicating factors.

##### Past experiences and outcomes

Participants described a range of previous experiences that informed their present and future decisions about accessing care. The most common accounts converged on encounters with health‐care providers. Two main subthemes were associated with driving the access experience and weighed heavily on participants' decisional processes: patient‐centred care and health‐care provider knowledge.

#### 2.5.4 Patient‐centred care

Participants' perceptions of the usefulness of a health‐care encounter were strongly influenced by whether the health‐care provider practised patient‐centred care. More specifically, they described three main components as being key to their overall experience: active communication and shared decision making, a holistic approach, and a respectful and empathetic provider. Participants positively described experiences when aspects of a patient‐centred approach were employed:

My family doctor has this really great way. You go with a symptom and he goes, ‘Okay, so you have three options, you can do physiotherapy or you can go on this medication, or you can get a knee replacement, sort of thing’. And he's like, ‘Which one sits well with you?’ And we'll discuss that. (Claire FG4)

Because she heard EVERYTHING I said. She asked me further questions, the things that have happened in my history. […] She talked to me like I was an intelligent human being. (Diane, FG1)

Participants also described many experiences with health‐care providers lacking in elements of patient‐centred care. Persons with MS wanted to be heard, to be listened to and to contribute to their management plan:

If you could find a practitioner who will hear you…that [acknowledges that] you do know your body, and listens. And then uses that information to help you, then that's, that's the goal. (Betty, FG1)

Participants placed importance on transparency of information, especially regarding available management options. Transparency was considered a crucial component of shared decision making, which most participants agreed was a necessity to effectively manage their MS.

I also found that treatment and management options were very narrow and even skewed. (Terry, FG3)

Participants' were highly concerned about being reduced to their MS, as many felt that once they were considered an ‘MSer’ there was no help to be received:

It was so easily, for me, personally a feeling of being put in the MS corner. You are of them. You should expect that […]. Somewhat implying, Why are you wasting my time? You should know better. (Luke, FG2)

One participant discussed how she strives to maintain independence, but that this effort had not been acknowledged by providers in the past:

Access to healthcare with MS means to keep my independence and to live a full life and be treated like a human being, and not just a person that's got MS so, ‘We can't help you’. (Sandra, FG 3)

Participants also wanted respectful and empathetic providers who would discuss the limitations of available interventions, given their personal situations. Salinda summarized this as:

Doctors who have no patience, or how about sympathy, empathy. Like, it's their job we get that, but it's our lives. (FG2)

When providers were not patient‐centred, multiple negative outcomes ensued. Participants described feeling disregarded, invalidated and dismissed. Ryan described feeling judged:

It looks like they're not even listening to me and they're saying, It's in your head. (FG4)

#### 2.5.5 Provider knowledge

Providers' MS‐related knowledge also impacted their access experiences. Participants understood that it was not possible for generalists to be specialized in MS; however, they expected them to know the basics and provide symptom management or referrals. Participants described multiple instances where they had negative experiences with family physicians who did not have the MS knowledge required to help them:

The Family [doctors], at the primary practice level, they're not educated [about MS]—They're not. And it's dangerous. (Samantha, FG1)

Participants also discussed that MS specialists lacked knowledge of the lived experiences and impacts of MS:

Just because they are knowledgeable in MS. […] They know the brain [but] they're not knowledgeable in an MS patient's day. (Charlotte, FG2)

Participants' past experiences of patient‐centred care and level of providers' MS‐related knowledge contributed to anticipated outcomes of seeking care. Negative past experiences and related poor outcomes led to the development of poor anticipated outcomes of future care. Diane explained that she no longer sought care because she anticipated no help:

And had no help. […] I got to the point where I had completely learned helplessness, […] that's why I stopped getting help, because I had tried. I tried and tried and tried, and there was none. And it's been very difficult. Being told you don't need help. (FG 1)

##### Complicating factors

Multiple additional complicating factors also weighed into the decisional process of whether or not to access care. The most common factors influencing their decisions were as follows: *availability*, *coordination of care, physical accessibility* and *affordability*.

A majority of participants reported that, in their experience, there are not enough MS neurologists in Canada, leading to low availability of these providers:

The availability of a neurologist. […] I had quite an experience trying to get one (Gail, FG2).

Participants described that the shortage of MS neurologists resulted in care not being timely. Many participants reported seeing their neurologist annually and in between, relying on other providers, or dealing with their MS‐related health issues on their own.

I go down there once a year, and it's a 15‐minute appointment, or maybe a half‐hour appointment. So, in one session, I'm supposed to tell them everything (laughs) that's been going on with me for a year. (Marie, TI)

Nicole described that due to low availability of MS specialists, she needed to depend on her family physician:

Requiring the services of a neurologically‐trained person, but being dependent on untrained family health team members, to your detriment. (FG 5)

Others described relying on multiple health‐care providers to receive their required care. Most considered this approach sub‐optimal as it required more energy to schedule and attend each appointment. Persons with MS shared experiences of getting lost between providers or receiving conflicting treatments from providers who did not communicate or coordinate care. Danielle highlighted this issue:

‘One of the biggest frustrations, to me, before I was stable, so following, a pretty serious relapse, was communication between different healthcare practitioners. […] to open dialogue between different providers’ (FG 1)

Availability of MS specialists was linked to the physical accessibility of health‐care services. Participants described MS clinics with MS trained specialists as only being located in larger cities. Rebecca from Northern Ontario described this situation:

I feel that we are alone in our illness up here. […] I would like to be in a place where I could go to a clinic. (TI)

Physical accessibility was also discussed in terms of environmental barriers in institutions and communities that hindered participants' ability to get to required health‐care services, particularly in the face of mobility issues and MS‐related fatigue. Paul stressed the importance of accessibility to overall access:

I'll gladly take two hours to go down to wherever I have to go if I know that, once I get there, I can get where I want to go you know, with, with some ease […] the accessibility is lacking. (FG2)

Closely linked to availability of services was affordability. Many participants found that available services funded through the conventional health‐care system did not always meet their needs. However, services that participants found impacted their quality of life, such as physical and occupational therapy, massage therapy and naturopathic medicine, which were not considered affordable:

Unfortunately, though, I'm limited by finances, so I can't get all that extra help that would be so [helpful]—it's difficult to get that extra help that I think is so vital to a person's health. (Krista, FG1)

Additional costs of accessing care were also described, for example, transportation, parking fees and lodging for out‐of‐towners. These costs were highest for individuals from northern communities, where flights and accommodations were needed. Individuals explained that costs were defrayed, but the process took months, and thus, it became untenable for those living on low incomes. Overall, many stated that the outcomes of care were simply not worth the energy, the time and the lasting fatigue:

Because it's so hard. […] It's a lot of work. It, it just, it's, you know. it's a lot of work.

[…] It's the energy it takes to go. Find parking at the [hospital], okay. I'm like, okay, I'll just deal with it. (Paul, FG2)

#### 2.5.6 The decision

After appraising their current health state as low to moderately threatening and weighing this appraisal against the accumulating negative past experiences and outcomes, and additional complicating factors, many participants decided that accessing health‐care was probably not worth it. Individuals described postponing health‐care access, and instead engaging in trial and error. In this transitory phase, participants attempted to self‐manage or deal with the health issue on their own or with peer support. One participant pointed out that she considers herself the best source of care:

The only, effective resource that I've ever had, consistently, is myself. Because I know how that person's going to respond and I know how to deal with her. (Danielle, FG1)

Many participants also sought complementary and alternative medicine, or simply waited to see if the issue worsened with time. Catherine described how she proceeded:

If it's something I can take care of myself, or something I had before and it's not that bad, I will take care of it myself. And then, if it doesn't work, then I'll seek care. Help, either my naturopath, or the ER if it's bad enough. (TI)

Participants also described searching for new providers that practised in a patient‐centred approach. Many discussed cycling through different family physicians and neurologists in order to find one practising this approach:

‘I've [had] one, two, three neurologists, two family doctors. Because the way I see it is that I have no choice to have this disease, but I have the choice who's going to help me’. (Amanda, FG2)

Other participants explained how they had lost trust in the conventional health‐care system and thus would prefer seeking help from complimentary services. Unfortunately, these services were cost‐prohibited, and thus, many disengaged from health‐care altogether. Participants in one focus group explained:

Samantha: […] I've had other things that I've gone for and I have had so little help that I actually gave up getting help. I didn't even bother going.

Veronica: I was going to say, about 15 years ago I kind of said there's no point.

Betty: […] no one helps. (FG1)

Throughout the trial and error phase of this decision‐making process, participants were continually reappraising their current health state. If their health state stabilized and the impact on their activity and participation was reduced, participants decided that accessing health‐care services was not worth it. If, however, participants appraised their health state as lasting or worsening, having a more pronounced impact on their daily functioning, or causing fears about their safety, then, at last, the answer to ‘Is it worth it?’ would be yes.

## 3 DISCUSSION

Experiences of access to health‐care, described by Ontarians with MS, were centred on the processes of seeking health care. These processes involved complex iterative decision making, informed by their experiences of living with MS, seeking and receiving health care, appraisal of health and threat perception, as well as environmental barriers. The key question driving this decision to access health‐care was ‘Is it worth it?’. Participants attempted to answer this question by evaluating if the energy and resources needed to pursue health‐care services were worth the anticipated outcomes. Most participants felt that accessing the conventional health‐care system was not worth it, until they were in a state of ‘crisis’ with no other options.

### 3.1 Findings in context

Interestingly, these findings show that persons with MS describe the process of seeking help for an MS‐related concern as central to their experience of access. This finding is consistent with newer conceptualizations of access to care^25^, ^26^ that integrate concepts from help‐seeking literature^24^, ^31^, ^32^. Our findings highlight the importance of bridging conceptualizations of access in health services research, often guided by the five As (affordability, accessibility, accommodation, availability and appropriateness)^33^, with social science research guided by the behavioural process of help‐seeking^24^, ^32^, ^34^. To understand the full experience of persons with MS, it is important to examine behavioural and environmental aspects of access beginning from the onset of a potential symptom^24^ to utilization of care^18^, ^33^. Our findings support the further application and evaluation of theories of access to health‐care that include help‐seeking as a component.

The findings also suggest that Ontarians with MS continue to experience unmet needs and dissatisfaction with health‐care services, which may be explained by their experiences of accessing health care. Studies examining access to care for people with MS have attributed unmet needs to traditional dimensions of affordability, accommodation and accessibility^5^, ^35^, ^36^. The participants in our study also described these dimensions of access; however, they emphasized the importance of patient‐provider interactions in fulfilling their care needs, consistent with research conducted in the UK^15^. Our participants placed importance on having a health‐care provider who was patient‐centred and knowledgeable about MS and its impact on daily activities. Patient‐centred care is described by the Canadian Medical Association as ‘seamless access to the continuum of care in a timely manner, based on need and not the ability to pay, that takes into consideration the individual needs and preferences of the patient and his/her family, and treats the patient with respect and dignity’^37^.

Participants explained having a chronic illness meant that their care needs and goals are often focused on improving quality of life and participation. Many participants felt that health‐care providers did not consider their preferences and goals or engage in bidirectional communication. These qualities have been listed as priorities by persons with MS^15^, ^38^ and individuals with other chronic illnesses^39^, ^40^. These experiences reflect a lack of patient‐centred care and are consistent with other qualitative work about health‐care experiences of persons living with chronic illness^40^, ^41^, ^42^. The tendency for health‐care providers to focus more on function and structure, as opposed to the desired, participatory outcomes left participants feeling unheard, de‐legitimized and un‐helped. This led many to engage in ‘doctor shopping’ until they found a provider practising in a patient‐centred approach that supported overall wellbeing. Many participants turned towards allied health or complementary health‐care providers^43^. Unfortunately, lack of funding for many of these services made them unrealistic as long‐term options, consistent with previous findings^5^, ^44^. The misalignment in care philosophies left participants with unmet needs and low satisfaction with care; therefore, overtime seeking help was not considered worth it. These findings suggest that ongoing efforts to support patient‐centred care^37^ are still needed.

By qualitatively exploring individual's selection of health‐care providers, we were able to ascertain the reasoning driving their selections. In addition to patient‐centred care, participants identified the importance of MS‐related knowledge. Generally, health‐care providers having the most MS‐related knowledge are MS‐specialist neurologists. Unfortunately, participants reported not having access to these providers due to low availability. The marked decrease in the neurological workforce^45^ and an increase in rates of MS^46^ in Canada highlight this problem. Low availability of specialized care led many participants to feel that they had to rely on their family physicians, who possessed little knowledge of MS. This concern has been previously reported in Canada and internationally^15^, ^35^, ^36^, ^47^, ^48^. Many participants felt that a heavy reliance on primary care was detrimental to their health and limited their management and treatment options. Previous work examining health‐care communication^40^ and provider preference^47^ among people with MS showed similar results.

### 3.2 What are the strengths and limitations?

A strength of this study is its focus on the perspectives and experiences of persons with MS related to engaging in the process of interacting with the health‐care system to gain access. Data were collected using focus groups and individual interviews which allowed for both breadth and depth of experiences. Our research team brings broad knowledge and experience in areas of disability and health policy, models of interprofessional team‐based care and primary care, and MS rehabilitation. Our combined background facilitated links across emerging ideas and enabled the team to challenge preconceptions about the factors influencing participant experiences (eg access to specialty care, geography, role of primary care, impact of disability and disease duration).

Potential limitations of the study are the sole focus on persons with MS residing in the province of Ontario and the high level of education within the sample. The recruitment process aimed for maximal variation by sampling from regions with differing levels of rurality and participants at different stages of MS; however, the strategies may have been more attractive to highly educated individuals. The focus on Ontario was due to time and cost constraints; however, by sampling across regions we hoped to heighten transferability. Although the variable size of the focus groups may have created differences in the level of engagement in sharing, careful analysis and comparison of findings across groups did not support this as a study limitation. Conducting longitudinal interviews may have allowed for a more robust view of changes in accessing health‐care overtime.

### 3.3 What are the next steps and practical implications?

Patient‐centred care is not a new concept within the Canadian health‐care system. The Charter for Patient‐centred Care was established in 2010, which is at the heart of health‐care reform^37^, ^49^. Our findings highlight the need to continue implementing strategies that improve the adoption of patient‐centred care, underlined by a biopsychosocial ideology^45^, ^49^. This will allow the health‐care system to respond more appropriately to the needs of persons living with MS^50^, especially, as the rates of MS and other chronic conditions rise in Canada^51^. Specifically, including outcome measures of quality of life, participation and independence to the care protocol of persons with MS may positively influence their satisfaction with care^36^.

To mitigate the low availability of MS neurologists, we should aim to equip family physicians with the appropriate MS‐related knowledge and skills to take on a larger role in the care of MS patients. Family physicians are well positioned to provide ongoing continuous care that is accessible and available^52^, and they are already commonly relied on by persons with MS^53^, ^54^. They could take on shared responsibility with neurologist to care for this patient population, as suggested by Oh et al, by supporting ongoing health maintenance, MS symptom and comorbidity management, and referrals to secondary services^55^. As Canada and Ontario move towards the adoption of patient medical homes, interprofessional team‐based, patient‐centred, continuous and comprehensive primary care^56^, the implementation of this shared care model^57^ becomes even more relevant, as it has the potential to address many of the concerns raised by persons with MS.

Comprehensive care for persons with MS should include MS‐related knowledge and patient‐centred care. MS Care Units, established in some European countries^58^, provide these features. MS Care Units are equivalent to funded cancer or stroke units that currently exist in Canada. This model of care is comprised of multidisciplinary teams that communicate and coordinate care plans to ensure all the patients' needs are met in one single appointment. These units are meant to function in a patient‐centred approach^58^ and posses the ability to incorporate affordable allied care, coordination and continuity of care, and MS knowledge, which encompass many factors that make seeking care worth it.

## 4 CONCLUSION

Multiple sclerosis is a complex, variable and unpredictable chronic neurological condition that requires lifelong management and care. The findings from this study suggest that persons with MS feel as though the energy requirements needed to overcome barriers to accessing health‐care services were not worth the outcomes of seeking the care. Patient‐centred care and health‐care provider knowledge were perceived as being pivotal yet oftentimes missing components of the current services available to them in Ontario. Findings suggest that a renewed effort to promote patient‐centred care with an underlying biopsychosocial approach to health‐care systems may improve persons with MS' experiences of health‐care services, reduce their associated avoidance and improve their quality of life.

## Data Availability

The data that support the findings of this study are available from the corresponding author upon reasonable request.
